# Investigation of polymer-derived Si–(B)–C–N ceramic/reduced graphene oxide composite systems as active catalysts towards the hydrogen evolution reaction

**DOI:** 10.1038/s41598-020-78558-x

**Published:** 2020-12-15

**Authors:** Quentin Hanniet, Moustapha Boussmen, Jonathan Barés, Vincent Huon, Igor Iatsunskyi, Emerson Coy, Mikhael Bechelany, Christel Gervais, Damien Voiry, Philippe Miele, Chrystelle Salameh

**Affiliations:** 1 Institut Européen Des Membranes, IEM, UMR 5635, Univ Montpellier, CNRS, ENSCM, 34095 Montpellier Cedex 5, France; 2 Laboratoire de Mécanique Et Génie Civil, UMR 5508, CNRS, Université de Montpellier, 34090 Montpellier, France; 3 NanoBioMedical Centre, Adam Mickiewicz University, Wszechnicy Piastowksiej str. 3, 61-614 Poznan, Poland; 4 Sorbonne Université, UMR 7574 CNRS, Laboratoire de Chimie de la Matière Condensée de Paris, LCMCP, 75005 Paris, France; 5 Institut Universitaire de France (IUF), MENESR, 1 rue Descartes, 75231 Paris Cedex 05, France

**Keywords:** Inorganic chemistry, Ceramics, Composites

## Abstract

Hydrogen Evolution Reaction (HER) is an attractive technology for chemical conversion of energy. Replacement of platinum with inexpensive and stable electrocatalysts remains a major bottleneck hampering large-scale hydrogen production by using clean and renewable energy sources. Here, we report electrocatalytically active and ultra-stable Polymer-Derived Ceramics towards HER. We successfully prepared ultrathin silicon and carbon (Si–C) based ceramic systems supported on electrically conducting 2D reduced graphene oxide (rGO) nanosheets with promising HER activity by varying the nature and the composition of the ceramic with the inclusion of nitrogen, boron and oxygen. Our results suggest that oxygen-enriched Si-B-C-N/rGO composites (O-SiBCN/rGO) display the strongest catalytic activity leading to an onset potential and a Tafel slope of − 340 mV and ~ 120 mV dec^−1^ respectively. O-SiBCN/rGO electrodes display stability over 170 h with minimal increase of 14% of the overpotential compared to ~ 1700% for commercial platinum nanoparticles. Our study provides new insights on the performance of ceramics as affordable and robust HER catalysts calling for further exploration of the electrocatalytic activity of such unconventional materials.

## Introduction

Thanks to its high energy density and environment-friendly nature, hydrogen is considered to be one of the most promising alternative energy carrier for the future^1^. Producing hydrogen in an efficient, sustainable and cost-effective way is prerequisite for the hydrogen economy. The most common method for producing hydrogen consists in natural gas reforming but such a process is responsible for the emission of greenhouse gases such as CO_2_. Alternatively, considerable efforts have been dedicated towards the development of electrocatalytic water splitting^2^. Platinum (Pt) remains the most active electrocatalyst for the Hydrogen Evolution Reaction (HER: 2H^+^  + 2e^−^ → H_2_) and is used in polymer electrolyte membrane electrolyzers. However, Pt is scarce and suffers from high-cost preventing its large-scale utilization^3^. Platinum is also unstable with time due to poisoning and leaching. Therefore, many electrocatalysts with a small onset potential and a low Tafel slope are currently being explored as alternatives to Pt^4–12^. In particular, low-dimensional materials have been identified as potential catalyst candidates for the electrocatalytic hydrogen evolution. 0D materials such as metal nanoparticles and 1D materials in the form of nanowires and urchin-like structures have been investigated for the HER^13^. As for 2D materials, transition metal dichalcogenides (TMDs) such as MoS_2_ have been the most examined^14^. Other 2D catalysts include doped graphene^15^, graphene-based materials^16–18^ and double-layer hydroxides^19^. The use of HER catalysts is however largely limited by their poor stability in acidic medium due to parasite reactions or leaching, while the recycling of metallic nanocatalysts remains difficult and costly.

Polymer-Derived Ceramics (PDCs) are a class of ceramics synthesized by the thermal decomposition of polymeric precursors that are best known for their excellent high-temperature stability, their remarkable thermo-mechanical properties and their chemical stability; attributed to their unique composition and microstructures^20,21^. Moreover, the final properties of PDCs can be modified in a controlled manner by tailoring the precursor’s composition and the processing parameters. The first generation of PDCs was the SiC fibers developed by Yajima in 1975 for high-temperature applications^22^. Since then, many other ternary and quaternary carbides and nitrides have been extensively investigated such as silicon oxycarbide (SiOC)^23^, boron modified silicon carbide (SiBC)^24,25^, silicon carbonitride (SiCN)^26^, boron and aluminum modified silicon carbonitride (SiBCN^27–29^, SiAlCN^21,30^). PDCs display semi-conducting behavior allowing them to be used in Micro-Electro-Mechanical Systems (MEMS)^31^, p–n junction devices^32^, micro glow plugs^33^ and pressure sensors^34^. The electronic structure of PDCs has however received little attention because of the amorphous nature of the materials complicating the theoretical calculations of their structure. The fact that PDCs have proved themselves eligible for electrochemical applications e.g. electrodes for lithium-ions batteries has motivated the exploration of PDC-based materials as potential electrocatalysts^35–39^.

Besides, several experimental and theoretical works have demonstrated that doping graphene or other nanocarbons with non-metallic heteroatoms e.g. oxygen, boron and nitrogen strongly enhances the HER^17,40^. Heteroatom doping can indeed tune the hydrogen adsorption strength on graphene in order to comply with the Sabatier principle which suggests that the free energy of hydrogen adsorption (ΔG_H*_) should be thermo-neutral^40^. The rational design of inorganic polymer precursors allows the preparation of PDCs with the inclusion of oxygen, nitrogen and boron atoms and therefore holds promises for the optimization of the HER activity of the ceramics.

Here we report the preparation of PDCs and graphene based composites as porous and ultra-stable electrocatalysts towards the production of hydrogen. The PDC/graphene composites were prepared by impregnating 3D reduced graphene oxide (rGO) macroporous foams with different ratios of SiCN and SiBCN preceramic polymer solutions in order to vary their porosity as well as their mechanical properties. The electrochemical activity towards HER was systematically investigated in acidic medium varying the PDCs content and composition. Our results show that PDCs are clearly HER active when supported on conducting graphene and the best HER performance is obtained in the case of O-SiBCN ceramics with an overpotential of ~ 580 mV *vs*. reversible hydrogen electrode (RHE) for a current density of 10 mA cm^−2^ and a Tafel slope of ~ 120 mV dec^−1^. Importantly, the PDC/graphene composites display stable HER activity over almost 170 h with a minimal increase of the potential at 10 mA cm^−2^ of only 14%. When testing under the same conditions, supported commercial platinum nanoparticles exhibit a tenfold increase of the overpotential highlighting the clear instability of the Pt catalyst. By taking advantage of the mechanical properties of the PDC/graphene composite, we demonstrate a free-standing electrode for the production of hydrogen that can sustain a current of 100 mA at an overpotential of 660 mV versus RHE. Together with the high stability and durability assessment, the electrochemical investigation was carefully conducted in order to correlate the catalytic activity to the nature, the porosity, the structure and the electrical conductivity of the composites. Our work sheds light on the electrocatalytic activity of inorganic ceramics as affordable and ultra-stable electocatalysts.

## Results and discussion

### Characterization of the PDC/rGO composites

**Figure 1:**
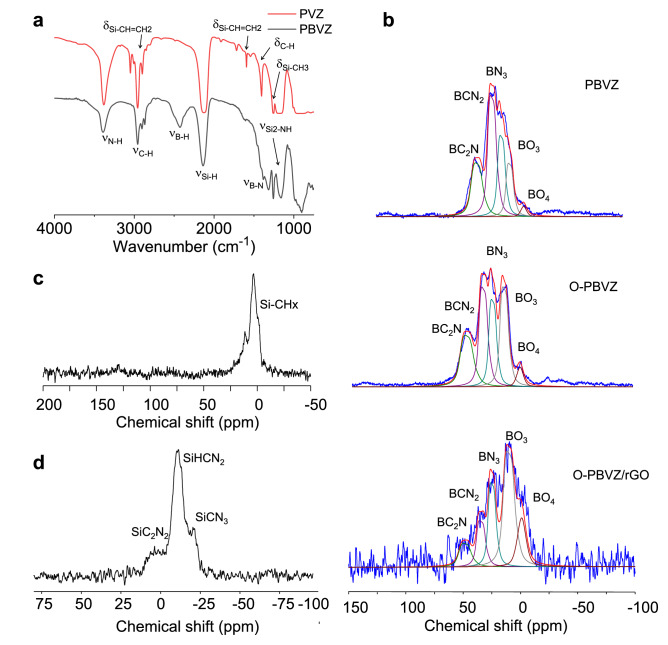
(**a**) FTIR spectra of PVZ and PBVZ preceramic polymers. (**b**) ^11^B MAS NMR spectra of PBVZ, O-PBVZ polymers and O-PBVZ/rGO composite. (**c**) ^13^C CP MAS NMR spectrum of PBVZ polymer and (**d**) ^29^Si MAS NMR spectrum of PBVZ polymer.

The chemical structure and the composition were first investigated for two preceramic polymers: PVZ (polyvinylsilazane) and PBVZ (boron-modified polyvinylsilazane) used as precursors of SiCN and SiBCN respectively. PVZ, a yellowish transparent liquid, is a commercial precursor of SiCN. We prepared the PBVZ polymer by treating PVZ with BDMS (borane dimethylsulfide complex) as a hydroboration agent^41^. We chose a controlled Si:B molar ratio equal to 3 to induce a complete hydroboration of the vinyl groups in the PVZ. The reaction between PVZ and BDMS also causes dehydrocoupling reactions between B-H from the BDMS and the N–H units present in the PVZ according to the general chemical formula of PVZ. We first used FT-IR spectroscopy to confirm that the reaction between PVZ and BDMS results in the formation of B–N and B–C bonds (Fig. 1a). The PVZ polymer displays the conventional absorption bands of the different chemical functions with notably the N–H bonds at 3382 cm^−1^ coupled to the vibration of Si–N at 1170 cm^−1^ ^42,43^. In the case of PBVZ, the intensities of the signals attributed to the vinyl groups decrease relative to those of PVZ suggesting thus that the hydroboration reaction occurred. In addition, the increase of the signal around 1325 cm^−1^ is associated to the emergence of B–N bonds via dehydrocoupling reactions between N–H of PVZ and B–H of the borane complex. The intensity of the peak of N–H bonds at 3382 cm^−1^ also decreased significantly after the incorporation of boron.

To get additional evidence of the formation of PBVZ, we performed solid-state NMR for identifying the local environment around boron, carbon and silicon atoms. The solid state NMR spectra of ^11^B, ^13^C and ^29^Si recorded for PBVZ are shown in Fig. 1b–d respectively. The ^11^B MAS NMR spectra are rather complex due to the heterogeneity in the local chemical environment of the boron nuclei and the presence of quadrupolar interactions. The presence of tri-coordinated boron atoms is however clearly observed. The signals can be fitted with components corresponding to ***B***C_2_N, ***B***CN_2_ and ***B***N_3_ (or also ***B***N_2_H since B-H unit is observed by FTIR) environments appearing at δ_iso_ ~ 55 ppm, 40 ppm and 30 ppm respectively in agreement with the literature^41^. These results thus confirm a dehydrocoupling reaction between B–H and N–H resulting in B–N. We note the presence of negligible amount of oxygen detected by the appearance of BO_3_ and BO_4_ environments at δ_iso_ ~ 15 ppm, and 0 ppm respectively. The ^13^C CP MAS NMR spectrum shown in Fig. 1c displays a signal centered around 10 ppm in the aliphatic region that can be attributed to the superposition of Si***C***H_3_ (~ − 2 ppm), Si***C***H_2_CH_2_-B (~ 6 ppm), Si***C***HCH_3_-B (~ 11 ppm) and SiCH_2_***C***H_2_-B (~ 16 ppm). The presence of these signals strongly supports the hydroboration reaction in perfect agreement with the FT-IR results. The ^29^Si MAS spectrum of the polymer in Fig. 1d displays a relatively complex distribution of SiN_x_C_4−x_ environments with the obvious presence of H-SiN_2_-CH_3_ units centered around − 20 ppm^44^. The two other peaks observed around − 5 ppm and − 30 ppm are attributed to SiC_2_N_2_ and SiCN_3_ respectively.

The beneficial role of oxygen as heteroatom in electrocatalysis was recently identified^45,46^. We prepared oxygen-rich PBVZ by exposing the preceramic polymer in air for 48 h to allow the diffusion of oxygen within the polymer and its reaction with the excess of B–H units. Similarly oxygen-rich preceramic polymers were prepared in order to produce O–SiC and O–SiCN. The modification of the C, Si and B environments was explored using NMR. The partial oxidation of PBVZ is confirmed by the increase of the BO_3_ and BO_4_ signals in the O-PBVZ compared to those in PBVZ (Fig. 1b). The oxidation of the SiN_x_ bonds into SiN_x_C_y_O_z_ is also identified at − 80 ppm as depicted in the ^29^Si MAS NMR spectrum (Fig. S1a). The ^13^C CP MAS NMR spectrum of O-PBVZ (Fig. S1b) is very similar to that of PBVZ. We also performed ^11^B MAS NMR on the O-SiBCN/rGO composite (Fig. 1b). Noticeably, the poor signal-to-noise ratio is explained by the conductive nature of rGO or the possible presence of free radicals preventing the correct tuning of the probe. Importantly, careful examination of the ^11^B spectrum reveals that signatures of the boron environment in the composite match those from the PBVZ preceramic polymer. O-SiBCN/rGO composite exhibits the same environments with nonetheless a higher proportion of ***B***O_3_ and ***B***O_4_ environments compared to ***B***C_2_N, ***B***CN_2_ and ***B***N_3_ confirming that mixing rGO and PDCs does not alter the chemical structure of the ceramic in the composite. Importantly, the absence of changes from the chemical environment of boron points out that the formation of the ceramics upon annealing does not induce any doping of the graphene support. Thermogravimetric analyses were performed on the different PDC/rGO composites in order to study their intrinsic thermal behavior during the polymer-to-ceramic conversion. The TGA curves display the weight loss of the PVZ, PBVZ and O-PBVZ when increasing the temperature up to 1000 °C under nitrogen (Fig. S2). Our results show that the addition of boron significantly decreases the weight losses of the PVZ improving the ceramic yield from 60 to 78% for PVZ and PBVZ respectively. We note that a high ceramic yield is strongly desired for the retention of the porosity, shape and structural properties of the impregnated rGO foam. Remarkably, the addition of oxygen does not drastically affect the weight loss of the PBVZ and the ceramic yield is maintained as high as 77%.

**Figure 2:**
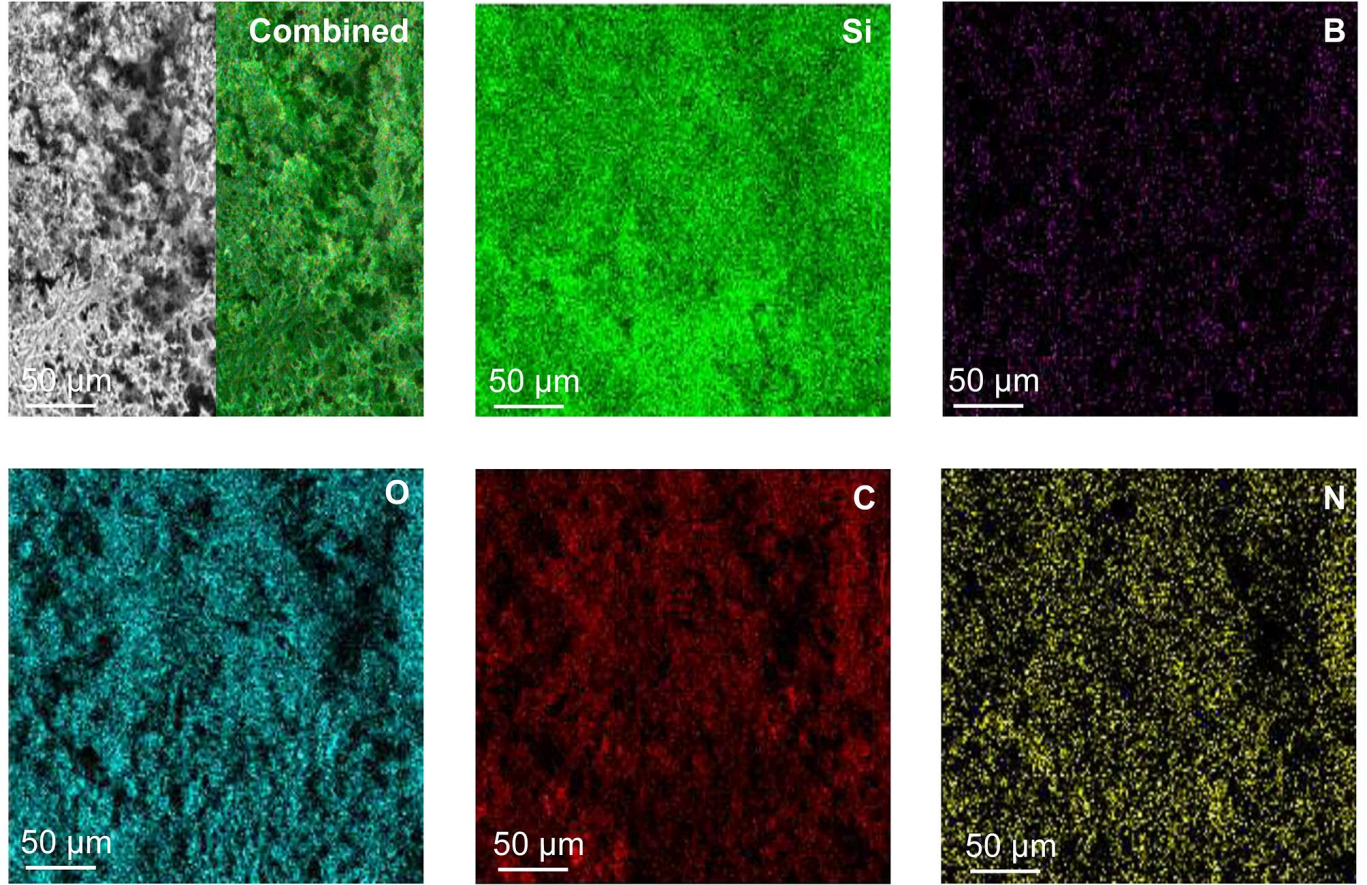
Low-magnification SEM image of O-SiBCN/rGO (top left panel) and the corresponding elemental maps.

**Figure 3:**
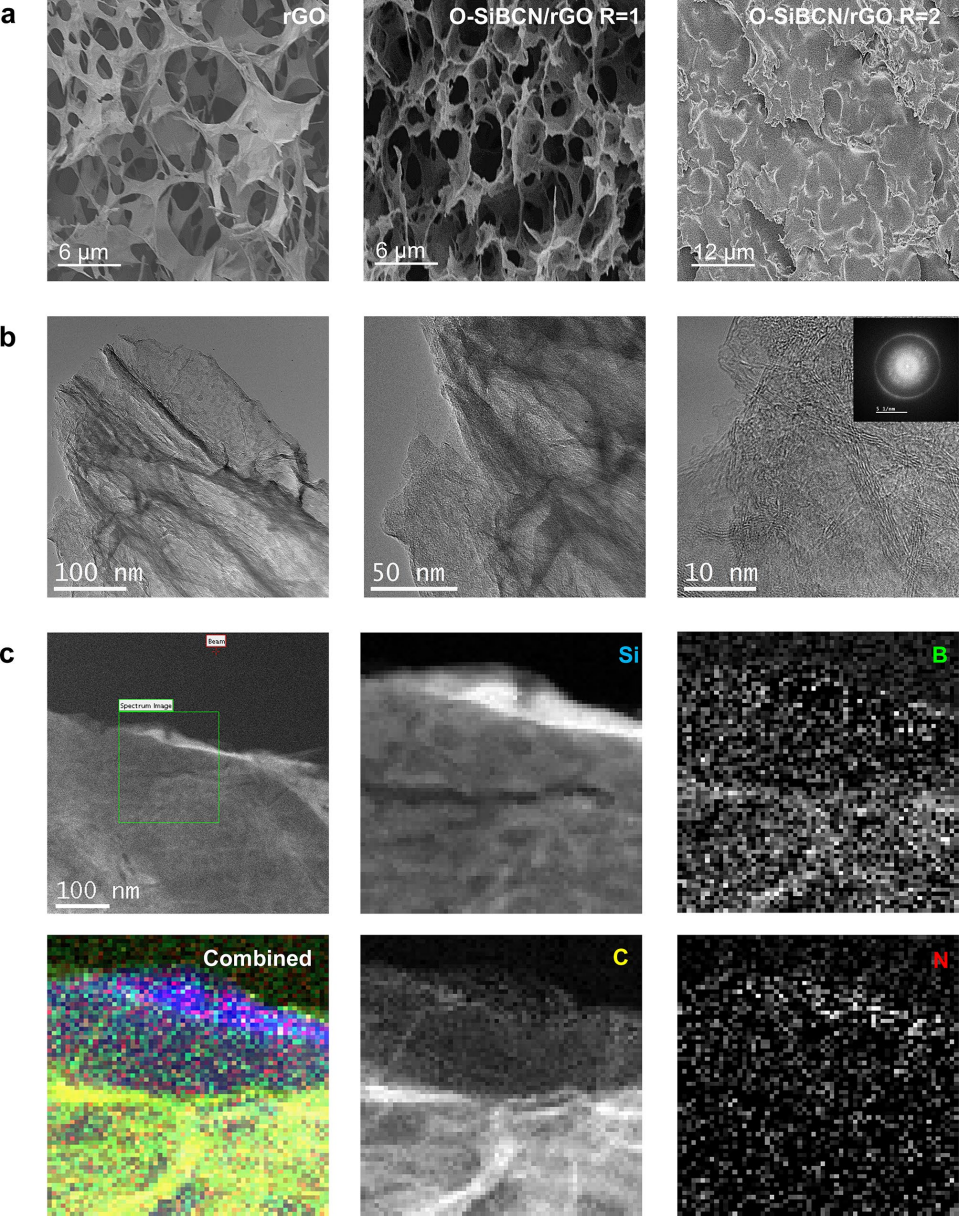
(**a**) High-magnification SEM images of rGO foam, O-SiBCN/rGO and O-SiBCN/rGO composite with a PDC/rGO ratio of 1 and 2 respectively. (**b**) TEM images of O-SiBCN/rGO (R_O-SiBCN/rGO_ = 1). (**c**) TEM images and the corresponding EELS chemical maps of O-SiBCN/rGO (R_O-SiBCN/rGO_ = 1) (Silicon (blue), Boron (green), Carbon (yellow), Nitrogen (red).

Next, we characterized the structure of the rGO and O-SiBCN/rGO composites using SEM and TEM. Figure 2 shows the low-magnification SEM images and the EDX elemental mapping of the O-SiBCN/rGO composite with a 1:1 ratio. The composite exhibits clear porosity, while the presence of B, N and Si suggests the successful infiltration of the ceramic inside the porosity of the rGO foam. High-magnification SEM images of rGO and O-SiBCN/rGO (Fig. 3a) confirm that the impregnation of rGO with the polymer occurred without altering the porous structure of the foam. This supports the fact that the pores are uniformly interconnected in the 3D composite and the foams retain their high porosity. Because of the macroscopic nature of our composite, we performed mercury intrusion porosimetry and we found a porosity of 93% for O-SiBCN/rGO *vs* 97% for rGO with an average pore diameter around 5 μm. The increase of the ceramic ratio in the composite leads to the progressive filling of the pores of the rGO scaffold (Figs. 3a and S3a). In order to further confirm the formation of ceramics on the rGO nanosheets, TEM and EELS observations were carried out on the composite (Fig. 3b,c). The two-dimensional nature is identified from the TEM images and attributed to rGO nanosheets. High-resolution TEM (HRTEM) images reveal that the wall of the foam consists of 4–8 rGO layers with sizes ranging from a 50 to few hundred nanometers (Fig. 3b). The interplanar distance (d_002_) for rGO is estimated to ~ 3.85 Å—close to interlayer distance of stacked graphene nanosheets of 3.43 Å. The presence of O-SiBCN on the top of rGO can be observed in Fig. 3c, while the EELS elemental mapping clearly confirms that all the components of the ceramic *i.e.* Si, B, C and N are present and homogenously distributed in the sample. We note that the diffraction pattern suggests a rather disordered structure of the composites as expected from the turbostatic stacking of the graphene nanosheets and the amorphous nature of the ceramic (Inset Fig. 3b). O-SiCN/rGO and O-SiC/rGO composites with a 1:1 ratio were also observed (Fig. S3b,c). Similarly to O-SiBCN/rGO, the porosity of the composite decreases with the increase of the PDC ratio (Fig. S3a). The presence of silicon carbide (from Si edge) and graphitic carbon (C edge) with small contents of amorphous carbon are detected from the EELS spectra.

**Figure 4:**
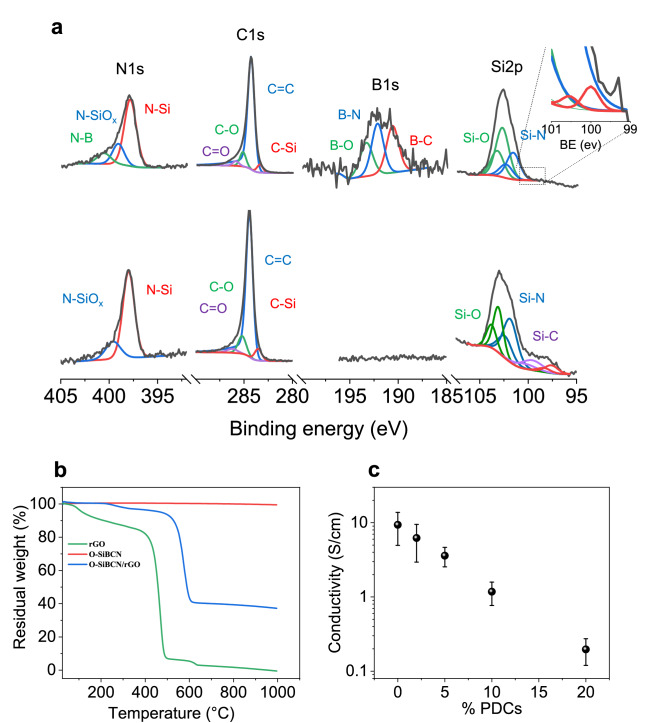
(**a**) XPS spectra of the N1s, C1s, B1s, Si2p regions of O-SiBCN/rGO (top) and O-SiCN/rGO composites (down). (**b**) TGA of the rGO foam, O-SiBCN ceramic, O-SiBCN/rGO composite under air up to 1000 °C and (**c**) electrical conductivity of PDCs/rGO composite systems.

X-ray photoelectron spectroscopy was then conducted to investigate the chemical composition of O-SiBCN and O-SiCN based composites pyrolyzed at 1000 °C (Figs. 4a and S4). The survey spectra of the composites confirm the presence of all the elements from the ceramics: Si, B, C, N and O, while no metals impurities can be detected (Fig. S4). High-resolution spectra of O-SiBCN/rGO from the core of the composites are presented in Fig. 4a. The intense peak at 284.4 eV is attributed to the C=C bonds from sp^2^ carbons in rGO. The peak at 285.2 eV suggests the presence of amorphous carbon while the existence of remaining C–O and C=O bonds comes from the incomplete reduction of the GO foams during the reduction step^47^. The Si–O, Si–N, N–SiO_x_ and N–Si peaks at 103.4 eV, 102.2 eV, 398.4 eV and 397.8 eV respectively confirm the existence of the SiN_x_O_y_ phase in the microstructure of the composite. The B1s and N1s regions can be deconvoluted with peaks at 190.7 eV, 192.3 eV and 193.4 eV from B–C, B–N and B–O respectively and N–B at 399.1 eV in agreement with the formation of the BN_x_O_y_ phase. Remarkably, the value of the Si:B ratio is estimated to 3—fully consistent with the expected Si:B ratio for O-SiBCN. This hints that no reaction occurred between the polymer and the rGO during the impregnation and the polymer-to-ceramic conversion process, strongly supporting the observations of NMR. The formation of SiCN ceramic in O-SiCN/rGO composites was also confirmed from the XPS analyses (Fig. 4a). Si–C, Si–N, and Si–O bonds are detected at 101.4, 102.4 and 103.7 eV respectively from the Si2p region, while the peaks of N–SiO_x_, N–Si, Si–N and Si–C are identified at 399.6, 398, 102.3 and 99.3 eV. XPS spectra of deconvoluted O1s are shown in Fig. S5.

To further confirm the presence of the ceramic in the composite, we performed TGA under air atmosphere (Fig. 4b). The weight loss at 1000 °C reaches 63% for O-SiBCN/rGO composites compared to 100% and 0.5% for rGO and O-SiBCN respectively. The weight loss for the composite is attributed to the combustion of rGO leaving the O-SiBCN ceramic as confirmed by our EDX analyses (Fig. S6). Overall, TGA and XPS confirm the presence of the ceramics within the different PDC/rGO composites. The electrical conductivities of the catalytic systems were examined for different ceramic ratios (Fig. 4c). We found that the conductivity rapidly decreases when increasing the ceramic ratio in the composite. For a ratio PDC:rGO equal to 1, the electrical conductivity is estimated to 713 S m^−1^ close to the conductivity of pristine rGO foam at ~ 1000 S m^−1^. This, combined with the high porosity of the PDC/rGO composites, is important for the fabrication of electrodes with high specific surface area.

### Electrocatalytic measurements

**Figure 5:**
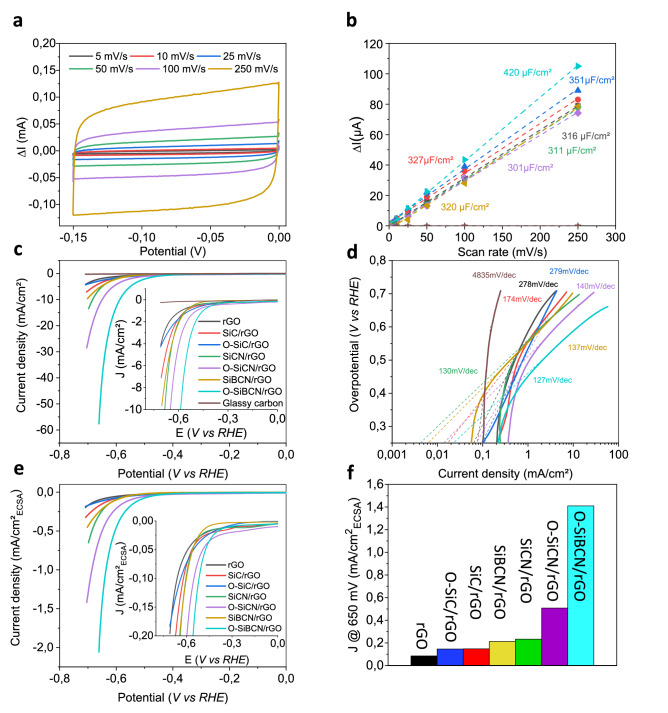
(**a**) Cyclic voltammetry curves for O-SiBCN/rGO composite. (**b**) Estimation of the double layer capacitance (C_dl_) for the different electrodes. (**c**,**d**) Polarization curves and Tafel slopes of rGO, SiC/rGO, O-SiC/rGO, SiCN/rGO, O-SiCN/rGO, SiBCN/rGO and O-SiBCN/rGO catalysts. (**e**) Corresponding ECSA-normalized polarization curves and (**f**) Histograms displaying the ECSA-normalized current density (**J**) at η = 650 mV for the different catalysts.

The electrocatalytic performance of the pristine ceramics and the rGO-based composites was investigated towards the evolution of hydrogen in 0.5 mol L^−1^ H_2_SO_4_. The HER activity from the glassy carbon was also measured to confirm that no activity arises from the electrode support. Figure 5 shows the electrochemical behavior of the different composite electrodes for a PDC:rGO ratio of 1—larger ratios of ceramics were also tested but do not lead to higher HER activity (Fig. S7). First, we estimated the electrochemical active surface area (ECSA) by measuring the double layer capacitance (C_dl_) of the composites (Fig. 5). The ECSA was calculated assuming an ideal capacitance of 0.015 mF cm^−2^ for a flat surface of graphene—note that the contribution of the ceramic can be neglected due to its insulating nature (Fig. S8)^48^. All the composites exhibit larger C_dl_ compared to the glassy carbon support revealing the porosity of the electrode in agreement with our SEM observations (Fig. 5b). The porous network combined with the high conductivity of the rGO scaffold provides (i) accessibility to more active sites and (ii) efficient transport pathways of the electrons to the active sites. Interestingly the composite electrodes display larger C_dl_ than pristine rGO (420 vs. 316 µF cm^−2^). This is attributed to the mechanical reinforcement of rGO by the ceramics that prevents the porous structure from collapsing. Of all the different PDC/rGO composites, O-SiBCN/rGO exhibits the largest C_dl_ and therefore the largest ECSA of 28 cm^−2^_ECSA_.

The polarization curves from the different composite catalysts i.e*.* SiC/rGO, O-SiC/rGO, SiCN/rGO, O-SiCN/rGO, SiBCN/rGO and O-SiBCN/rGO are presented in Fig. 5c. The HER activity is clearly increased in the case of the PDC/rGO compared to glassy carbon and rGO alone—ruling out any possible activity from impurities present in rGO or the conducting support. To examine the role of rGO on the catalytic performance, we assess the electrochemical behavior of the ceramics alone (Fig. S8a,b). We can see that the ceramics display poor HER activity demonstrating that rGO allows activating the PDCs via a synergistic effect as previously described for supported semiconducting materials^49^.

Remarkably O-SiBCN/rGO demonstrated the best performance among the different PDC/rGO electrodes with an onsetpotential of -340 mV *vs.* RHE and a current density as high as 60 mA cm^−2^ at an overpotential of η = 660 mV. O-SiBCN/rGO also exhibits the lowest Tafel slope at 127 mV dec^−1^ suggesting faster kinetics and the overpotential at 10 mA cm^−2^ reaches 590 mV (Fig. 5c). For comparison, overpotentials of η = 51 mV and η = 20 mV for driving a current of 10 mA cm^−2^ have been measured using respectively Pt-based alloyed catalysts such as Pt–Pd supported on graphene oxide and Pt–Ni–Co^50,51^. As for noble metal-free catalysts such as phosphides, overpotentials of 130–200 mV are requested to produce a current of 10 mA cm^−2^ ^52^; comparable to that of MoS_2_ deposited on graphene^49^. The decrease in Tafel values for the composites compared to their ceramic counterparts emphasizes the role of rGO as a conductive support. It has been shown that a Tafel slope value of 120 mV dec^−1^ corresponds to a kinetically limiting stage attributed to the hydrogen adsorption: Volmer step, H^+^  + e^−^ $\to$ H* ^53^. In the case of O-SiBCN/rGO, the value of 127 mV dec^−1^ suggests that the reaction on the catalyst surface proceeds via the Volmer-Heyrovsky mechanism with the Volmer step being the rate determining step (RDS). To obtain a fair comparison of the electrodes and avoid any contribution from the porosity of the different composites, we normalized the geometrical current density to the ECSA (Fig. 5e). We estimated the ECSA-normalized current density at an overpotential of η = 650 mV to compare the different composites and the performances follow the trend: rGO < O–SiC/rGO ≃ SiC/rGO < SiBCN/rGO ≃SiCN/rGO < O-SiCN/rGO < O-SiBCN/rGO (Fig. 5f).

Based on our electrochemical measurements and the physical characterization of the composite, we speculate that the active sites in the composite O-SiBCN/rGO are likely to be located at BN_3_, BC_x_N_y_ sites of the ceramic. This calls for additional investigation notably using first principle calculations that are however beyond the scope of this paper.

**Figure 6:**
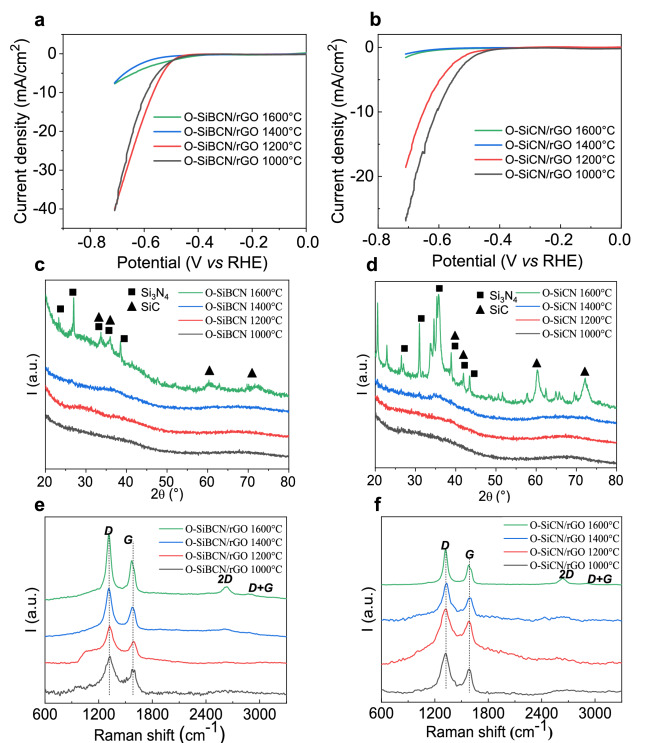
Polarization curves of O-SiBCN/rGO (**a**) and O-SiCN/rGO (**b**) composites at different temperatures. (**c**,**d**) X-Ray diffractograms of O-SiBCN ceramics and O-SiCN ceramics at different temperatures. (**e**,**f**) Corresponding Raman spectra of O-SiBCN/rGO and O-SiCN composites.

To get further understanding of the nature of the active sites of the O-SiBCN ceramics, we investigated the effect of the crystallinity of the ceramics on the electrocatalytic activity of O-SiBCN/rGO and O-SiCN/rGO by annealing the composites at increasing temperatures (Fig. 6a,b). Other amorphous catalysts such as metal chalcogenides, metal alloys, metal phosphides have already demonstrated larger catalytic activity relative to their crystalline counterparts^54–56^. Both O-SiBCN and O-SiCN catalysts remain active after annealing at temperatures up to 1200 °C but then lose most of the activity at 1400 °C and above. To elucidate the origin of the activity loss, the PDCs were characterized using X-ray diffraction and the PDC/rGO composites using Raman spectroscopy. Both O-SiBCN and O-SiCN ceramics progressively crystallize at temperature higher than 1200 °C as shown by the rise of the peaks at 35.7° (111), 60.3° (220) and 71.25° (311) corresponding to the formation of nanosized β-SiC crystals (Fig. 6c,d). These peaks are more obvious in the case of O-SiCN (Fig. 6d). This is attributed to the presence of boron within the ceramic that delays the crystallization of Si–B–C–N systems compared to Si–C–N ones. The incapacity of Si_3_N_4_ to react with free carbon via the carboreduction reaction: Si_3_N_4_ + 3C $\to \hspace{0.17em}$SiC + 2N_2_ primarily induces the crystallization into β-Si_3_N_4_ as confirmed by the peaks detected at 26.5, 33.7° and 36.1°^57^. Of note, the presence of oxygen has no deleterious effect on the amorphous behavior^57^ which may suggest that the selected ceramics SiBCN and O-SiBCN share similar microstructural framework. The increase of the composites crystallinity is also confirmed by the strengthening of Raman signatures from rGO graphitic domains (Fig. 6e,f). The position of the G band of O-SiBCN/rGO pyrolyzed between 1000 °C and 1600 °C continuously blue-shifts from 1587.5 to 1579.7 cm^−1^ due to the transition from amorphous disordered carbon to graphitic domains in agreement with the increase of the 2D band^58^. Overall our results point out the importance of the amorphous ceramics—notably O-SiBCN—on the electrocatalytic activity towards the production of hydrogen.

**Figure 7:**
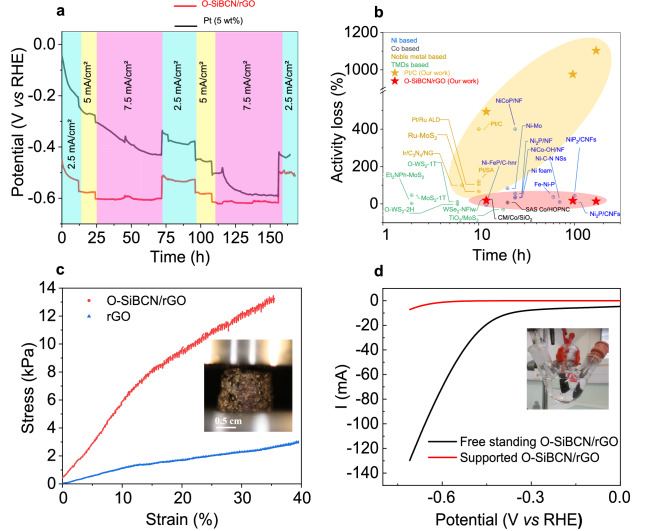
(**a**) Chronopotentiometry measurements for supported Pt/C electrodes and O-SiBCN/rGO composite over 168 h. (**b**) Change in overpotential (%) for O-SiBCN/rGO and other electrocatalysts in the literature: (Ni-FeP/C-hnr^62^ (hnr = hallow nanorod), Ni_2_P/CNFs^63^ (CNFs = carbon nanofibers), NiP_2_/CNFs^63^, Ni foam^64^, NiCo-OH/NF^64^ (NF = nickel foam), Ni_2_P/NF^64^, NiCoP/NF^64^, Ni-C-N NSs^65^ (NSs = nanosheets), Ni-Mo^66^, Fe–Ni-P^67^, CM/Co/SiO_2_^68^ (CM = carbon material)_,_ SAS/Co/HOPNC^69^ (SAS = single-atomic Co sites, HOPNC = embedded in hierarchically ordered porous N-doped carbon), Ir/C_3_N_4_/NG^70^, Pt/Ru ALD^71^ (ALD = atomic layer deposition), Pt/SA^71^ (SA = single atomic), Pt/C^71^, Ru-MoS_2_^72^, MoS_2_ 1T^73^, Et_2_NPh fct MoS_2_^73^, TiO_2_/MoS_2_^74^, O-WS2-1T^75^, O-WS2-2H^75^, WSe_2_ NFlw^76^ (NFlw = nano flowers). (**c**) Compression curves of the rGO foam and the O-SiBCN/rGO composite. The inset shows that the composite remains stable after compression. (**d**) Comparison of the polarization curves from the O-SiBCN/rGO composite catalyst deposited on the glassy carbon electrode and free-standing O-SiBCN/rGO. Note that the potentials of the free-standing electrode were not iR loss corrected.

To further explore the potential of the O-SiBCN/rGO composites, we studied the stability of the HER activity in acidic medium. The electrode was continuously subjected to various current densities for a total of 168 h. During the stability test, the applied current density was set to 2.5 mA cm^−2^, 5 mA cm^−2^ and 7.5 mA cm^−2^ for 48, 24 and 96 h, respectively. The modulation of the requested current density aimed at simulating the variation of the supply power similarly to the case of an electrolyzer connected to a photovoltaic cell. For a fair comparison commercial 5%Pt/C electrode was subjected to the same procedure (Fig. 7a). As expected commercial Pt nanoparticles initially show better HER performance as shown by the low overpotential of 51 mV at 2.5 mA cm^−2^—smaller than in the case of O-SiBCN/rGO of 454 mV. Remarkably, the overpotential for Pt rapidly increases by 493% during the first 12 h suggesting a rapid loss of activity. This is likely due to surface poisoning of the Pt surface by impurities from the electrolyte solution as well as possible leaching of the Pt nanoparticles in the acid electrolyte. Conversely, O-SiBCN/rGO exhibits significantly better retention of the HER performance over ≃ 170 h with an increase of the overpotential of 18.4%, 0.3% and ≃ 0% after 12, 24 and 48 h at 2.5 mA cm^−2^, 5 mA cm^−2^ and 7.5 mA cm^−2^, respectively. After 168 h, the difference of the overpotential between O-SiBCN/rGO and 5%Pt/C sharply decreases from 424 mV to only 85 mV. These results reveal the interest of O-SiBCN/rGO composite as an ultra-stable electrocatalyst for the HER in acidic medium. We also compared the stability of O-SiBCN/rGO electrode with that of several electrocatalysts reported previously in the literature. Our results clearly show outstanding stability of O-SiBCN/rGO over a long period of time demonstrating thus the potential of such materials towards realistic operation (Fig. 7b).

To demonstrate the advantage of the PDC/rGO composites for electrocatalysis, the mechanical properties of O-SiBCN/rGO and pristine rGO foams were evaluated by compression tests. The Young’s moduli for both pristine and PDC/rGO composites were deduced from the first linear part of stress–strain curves (Fig. 7c). Our results show that the O-SiBCN/rGO and rGO samples have a plastic behavior with a plasticity cross-over around 13% and 11% strain respectively. The Young’s modulus of O-SiBCN/rGO is estimated to almost 50 kPa—approximately fourfold larger than that of rGO. This considerable improvement of the stiffness of the composites is attributed to the presence of the ceramic on the rGO nanosheets. The plasticity modulus, extrapolated from the second linear part of the strain–stress curves, also decreases from 50 kPa for rGO to 25 kPa for O-SiBCN. Our mechanical investigations thus reveal that the controlled coating of the rGO foam with the ceramics strongly benefits the mechanical properties of the composite and allows the preparation of mechanically-robust electrodes. To validate our concept, we fabricated a free-standing electrode based on O-SiBCN/rGO. Thanks to the mechanical properties of the ceramics reinforcement and the electrical conductivity of the rGO scaffold, the electrode can directly be used without any conducting support. Such rational design of the composite electrode demonstrates a high porosity thanks to the rGO nanosheets, while the HER activity originating from the O-SiBCN ceramic allows reaching a current of almost 130 mA (Fig. 7d).

## Conclusion

Ceramics have been largely disregarded in electrocatalysis because of their insulating behavior. Here, we demonstrated an example of engineered PDC/rGO based composites as electrocatalytic active materials. Within the composite, rGO acts as a porous conductive network, with a high porosity of 93% and a high electrical conductivity allowing fast transport of the charge carriers and consequently activating the supported ceramic. In-depth investigations revealed that the HER activity of the ceramics can be modulated by the presence of O, N and B in the Si–C systems. Our careful measurements based on the ECSA and linear voltammetry allowed identifying O-SiBCN ceramic as stable HER catalyst. O-SiBCN/rGO exhibits the best performance with an onset potential of − 340 mV vs*.* RHE, while exhibiting an outstanding stability over almost 170 h with a minimal increase of the overpotential. Importantly, our results demonstrate the potential of ceramics for electrocatalysis when prepared in the form of an ultrathin film deposited on a conducting substrate. Considering the great stability of ceramics, we believe our work opens perspectives in the rational design of ceramic/graphene composites with controlled composition and rational structure and sheds light on novel applications of inorganic ceramics in electrocatalysis.

## Methods

### Reagents and materials

Polyvinylsilazane (**PVZ, also called** durazane)—a commercial preceramic polymer for SiCN—was purchased from Merck. AllylHydridoPolyCarboSilane (SMP-10, abbreviated **AHPCS**)—a commercial preceramic precursor for SiC—was purchased from Starfire. Toluene (anhydrous 99.8%, Sigma Aldrich), borane dimethylsulfide complex (BDMS, 2 M in toluene under argon, Alfa Asear), Nafion (D-520 dispersion, 5%w/w in water and 1-propanol, Sigma Aldrich), isopropanol (100%, VWR chemical), potassium permanganate (KMNO_4_, 99.0%min., Alfa Aesar), hydrogen peroxide (H_2_O_2_, ACS, 29–32% aqueous solution, Alfa Aesar) and sulfuric acid (H_2_SO_4_, ACS reagent 95–98%, Sigma Aldrich) were used as received without further purification.

### Materials synthesis

#### Synthesis of the preceramic polymer precursor of SiBCN

All chemicals and synthesis steps were handled in an argon filled glovebox (MBraun, MB-Labstar, O_2_ and H_2_O < 0.5 ppm) and using Schlenk techniques on a vacuum/argon line (with argon purified through a Siccapent phosphorous pentoxide column and BTS catalyst). PVZ and BDMS reacted through hydroboration and dehydrocoupling reactions in a quantitative yield leading to a boron-modified polyvinylsilazane abbreviated as **PBVZ**. BDMS was added dropwise at 0 °C into a three-neck round-bottom flask containing PVZ and toluene under stirring. When the addition was complete, the mixture was left under stirring at RT for 3 days. The solvent was then removed by distillation at reduced pressure. A moisture and air-sensitive glass-like white polymer was obtained with a yield of 81 wt%. The same procedure was applied to synthesize the precursor of SiBC by reacting AHPCS with the borane complex yielding also a **B-AHPCS** glass-like white polymer.

#### Synthesis of rGO foams

In a first step, GO was synthesized according to a modified Hummers method starting from graphite powder^59^. In a typical synthesis, the graphite was oxidized by concentrated sulfuric acid and potassium permanganate. Deionized water was slowly added, followed by hydrogen peroxide. GO was then washed with deionized water and subsequently centrifuged several times until a concentrated solution with a neutral pH was obtained. GO foams were synthesized by hydrothermal synthesis; where aqueous solutions of GO (1.5 mg mL^−1^) were introduced in an autoclave (Parr instrument company) and heated at 180 °C for 12 h. The as-formed wet gels were then fully frozen and consequently freeze-dried (Labonco Freezone 4.5; 0.05 mBar) yielding 3D GO foams. The reduction of the GO into reduced graphene oxide (rGO) foams was carried out by thermal treatment at 900 °C for 2 h under reducing atmosphere (Varigon, 5%H_2_/Ar) in a Vecstar tubular furnace.

#### Preparation of PDC/rGO composites

PDC/rGO composites were prepared via a vacuum-assisted impregnation process in a Schlenk-type flask. The preparation of SiCN/rGO is described hereafter as an example. The preceramic precursor i.e. PVZ was dissolved in toluene under stirring and added to a rGO foam. rGO was previously dehydrated at 120 °C under vacuum for 12 h to remove all the trapped air and moisture between the pores of the foam. The mixture was allowed to infiltrate the pores of the foam by capillarity under static vacuum for 24 h. Different ratios between the preceramic precursors and the foams were prepared varying from 1 to 10 in weight. After washing with toluene (4 $\times \hspace{0.17em}$10 mL) and drying, the PVZ/rGO composite was then transferred into a quartz tube in a tubular furnace (Nabertherm type RS 80/500/11 Germany) and subjected to a pyrolysis under nitrogen at 1000 °C for 2 h with a heating rate of 1 °C min^-1^ and a cooling rate of 2 °C min^−1^. The slow heating rate generates nanoporosity within the ceramic enhancing thus the surface area as previously described^60^ while avoiding the collapse of the composite structure. This thermal treatment leads to the ceramic conversion (PVZ → SiCN) and further reduces the rGO in order to have a chemical structure as close as possible to graphene. Other PDC-based composites were prepared accordingly with the following compositions: SiBCN/rGO, SiC/rGO and SiBC/rGO by using PBVZ, AHPCS and B-AHPCS as preceramic polymers respectively.

#### Crystallization of PDC/rGO composites

The PDC/rGO composites were subsequently annealed at high temperatures (1200–1600 °C) for 2 h under nitrogen in a graphitic furnace (Gero, Model HTK8) in order to induce crystallization of the ceramics. During all the thermal treatments, the materials were loaded in the furnace, after what the furnace chamber was evacuated for 1 h then filled with nitrogen with a 200 mL.min^-1^ flow rate during the treatment.

### Materials characterization

The chemical structure of the preceramic polymers was characterized by Fourier Transform InfraRed (FT-IR) spectroscopy with a Nicolet iS50 Thermo scientific spectrometer using KBr pellets (2 wt% of polymer mixed with previously dried KBr powder followed by compaction into a dense pellet). It was also characterized by solid-state NMR spectroscopy. Solid-state ^13^C CP MAS and ^29^Si MAS NMR were recorded on a Bruker Avance 300 spectrometer (B_0_ = 7.0 T, ν_0_ (^1^H) = 300.29 MHz, ν_0_ (^13^C) = 75.51 MHz, ν_0_ (^29^Si) = 59.66 MHz) using a 4 mm Bruker probe and spinning frequency of 10 kHz. While ^11^B MAS spectra were recorded on a Bruker Avance 700 spectrometer (B_0_ = 716.4 T, ν_0_ (^11^B) = 224.6 MHz) using a 3.2 mm Bruker probe and spinning frequency of 20 kHz. ^11^B spectra were acquired using a spin-echo θ–τ–2θ pulse sequence with θ = 90°, to overcome problems related to the probe signal. The τ delay (50 μs) was synchronized with the spinning frequency. The chemical shift values were referenced to TMS for ^13^C and ^29^Si, and BF_3_.OEt_2_ for ^11^B. The NMR spectra were simulated with the DMFIT program^61^.

Thermogravimetric analysis (TGA) was carried out on a TGA-STD Q600 thermal analysis device under atmospheric pressure from RT up to 1000 °C with a heating rate of 5 °C.min^-1^. TGA was performed under nitrogen in the case of the preceramic polymers to investigate their ceramic conversion and under air for the PDC/rGO composites to study the effect of the thermal annealing in a reactive atmosphere. Scanning Electron Microscopy (SEM) (Hitashi S4800, operating with an acceleration voltage between 0.1 kV and 30 kV) and Transmission Electron Microscopy (TEM) measurements (JEOL ARM 200F operating at 200 eV, equipped with an energy dispersive X-Ray spectroscopy—EDX and an energy electron loss spectroscopy—EELS) were used to investigate the morphology and microstructure of the composites. Energy dispersive X-ray spectroscopy (Detector: Oxford Instruments X-Max ^N^ SDD; Microscopy: Zeiss EVO HD15) and X-ray photoelectron spectroscopy XPS (ESCALAB 250, ThermoElectron equipped with an Al Kα line 1486.6 eV as monochromatic excitation source with a 400-μm spot size) were used to analyze the chemical composition of the ceramics and composites. The samples for XPS were prepared on a silicon wafer coated with gold. Raman spectroscopy (LABRAM 1B) and X-ray diffraction (Philips X’pert PRO X-ray diffraction system operating at 20 mA and 40 kV from 10 to 90° with a step size of 0.0167, using a Kα1of copper as source) were used to study the evolution of the structure of the PDCs with and without rGO with the temperature. The electrical conductivity was measured by using a two-probe method with silver as contact metal. Compression measurements were carried out on the rGO foam and on the O-SiBCN/rGO composite using a LLOYD 01/LF compression device equipped with a 50 N capacity force sensor. The porosity of the foams was characterized by mercury intrusion porosimetry using a Micromeritics Autopore IV 9500 penetrometer.

### Electrochemical measurements

Electrochemical characterizations were performed in a traditional three-electrode setup using a VSP500 potentiostat from Biologic in a 0.5 mol L^−1^ H_2_SO_4_ electrolyte solution. A Hg_2_Cl_2_/Hg electrode and graphite rod (Alfa Aesar) were used as the reference and the counter electrodes respectively. The working electrodes were prepared by dispersing 5 mg of the catalyst in 1.0 mL of deionized water, isopropanol and 5 wt% Nafion solution (at 62.5; 25 and 12.5 vol%, respectively). 10 µL of the ink was loaded onto a glassy carbon (Sigradur) electrode with 0.2 cm^2^ active area corresponding to a weight density of 1.25 mg cm^−2^. All potentials were corrected to the reversible hydrogen electrode (RHE). The electrochemical impedance spectroscopy has been recorded at 550 mV vs. RHE with an AC voltage of 5 mV from 10^6^ to 0.01 Hz. The double-layer charge capacitance (C_dl_) was measured by cycling the electrodes at increasing steps from 5 mV s^−1^ up to 250 mV s^−1^ between 241 and 91 mV *vs*. RHE. The C_dl_ for the different electrodes was estimated from the slope of the anodic and cathodic current densities at 166 mV *vs*. RHE. All potentials presented were corrected for iR losses unless specifically noted.

## Supplementary information

Supplementary Information 1.
