# Supplementary material for: Investigation of polymer-derived Si–(B)–C–N ceramic/reduced graphene oxide composite systems as active catalysts towards the hydrogen evolution reaction
Source: Sci Rep. 2020 Dec 15;10:22003. doi: 10.1038/s41598-020-78558-x (PMC7738544; doi:10.1038/s41598-020-78558-x)
Supplement: Supplementary file 1 — Supplementary Information 1. [file 41598_2020_78558_MOESM1_ESM.pdf]

Supplementary material for

Investigation of Polymer-Derived Si-(B)-C-N ceramic/reduced graphene oxide composite systems as active catalysts towards the hydrogen evolution reaction

Quentin Hanniet[1], Moustapha Boussmen[1], Jonathan Barés[2], Vincent Huon[2], Igor Iatsunskyi[3], Emerson Coy[3], Mikhael Bechelany[1], Christel Gervais[4], Damien Voiry[1], Philippe Miele[1,5*], Chrystelle Salameh[1*]

Affiliations:

[1] *Institut Européen des Membranes, IEM, UMR 5635, Univ Montpellier, CNRS, ENSCM, 34095 Montpellier Cedex 5, France*

[2] *Laboratoire de Mécanique et Génie Civil, UMR 5508 CNRS-Université de Montpellier, 34090, Montpellier, France*

[3] *NanoBioMedical Centre, Adam Mickiewicz University, Wszechnicy Piastowksiej str. 3, 61-614 Poznan, Poland*

[4] *Sorbonne Université, UMR 7574 CNRS, Laboratoire de Chimie de la Matière Condensée de Paris, LCMCP, F-75005 Paris, France*

[5] *Institut Universitaire de France (IUF), MENESR, 1 rue Descartes, 75231 Paris Cedex 05, France*

Corresponding authors: chrystelle.salameh@enscm.fr *(Chrystelle Salameh)*

philippe.miele@umontpellier.fr *(Philippe Miele)*

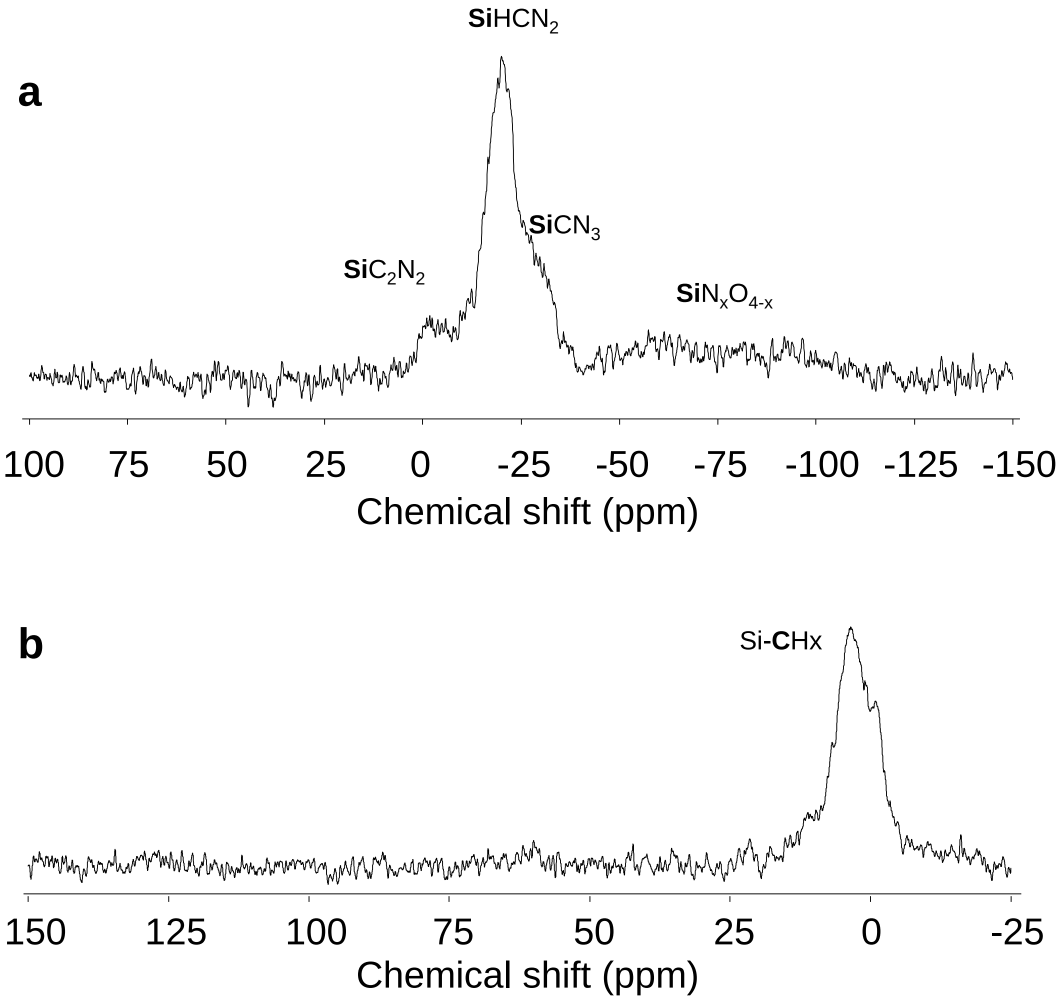

Figure S1: (a) ^{29}Si MAS and (b) ^{13}C CP MAS NMR spectra of O-PBVZ polymer.

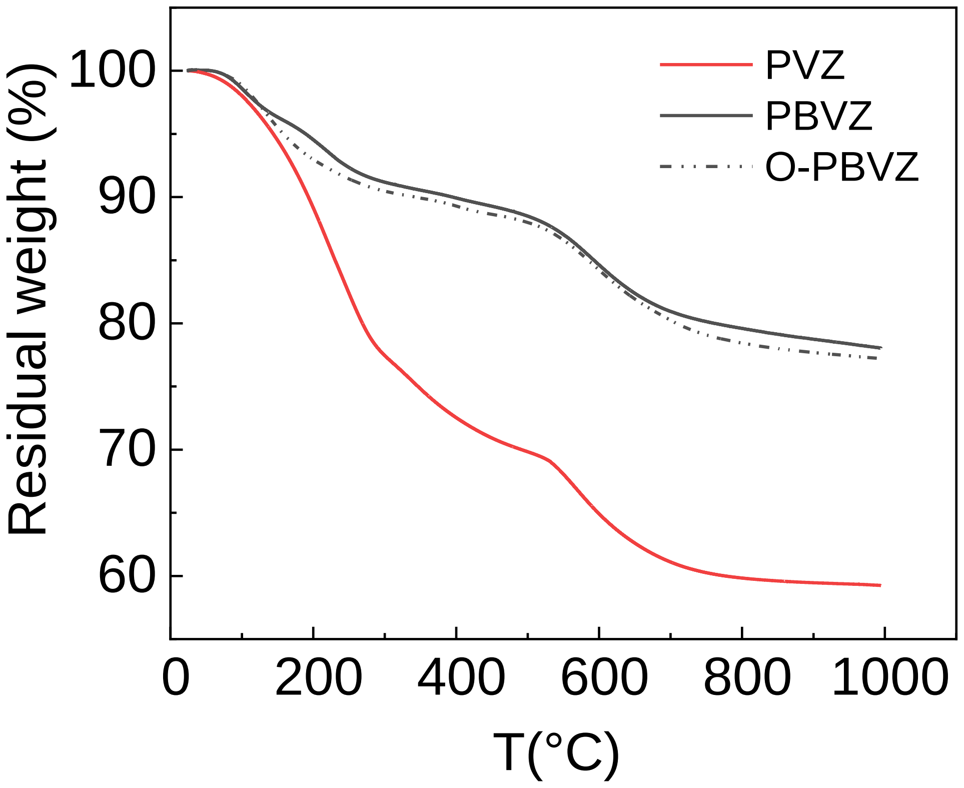

Figure S2 : TGA curves of PVZ, PBVZ and O-PBVZ polymers up to 1000°C under N_2 (heating rate 5°C.min^{-1}). The polymer-to-ceramic transformation occurs through several distinctive stages, in particular, two weight losses are predominant: the first one from room temperature up to 300°C (~3 wt.%) and the second one from 300 up to 1000°C (~19 wt.%). They are associated to the decomposition of organic components accompanied by the release of gaseous compounds such as H_2 and CH_4 and other volatile species.

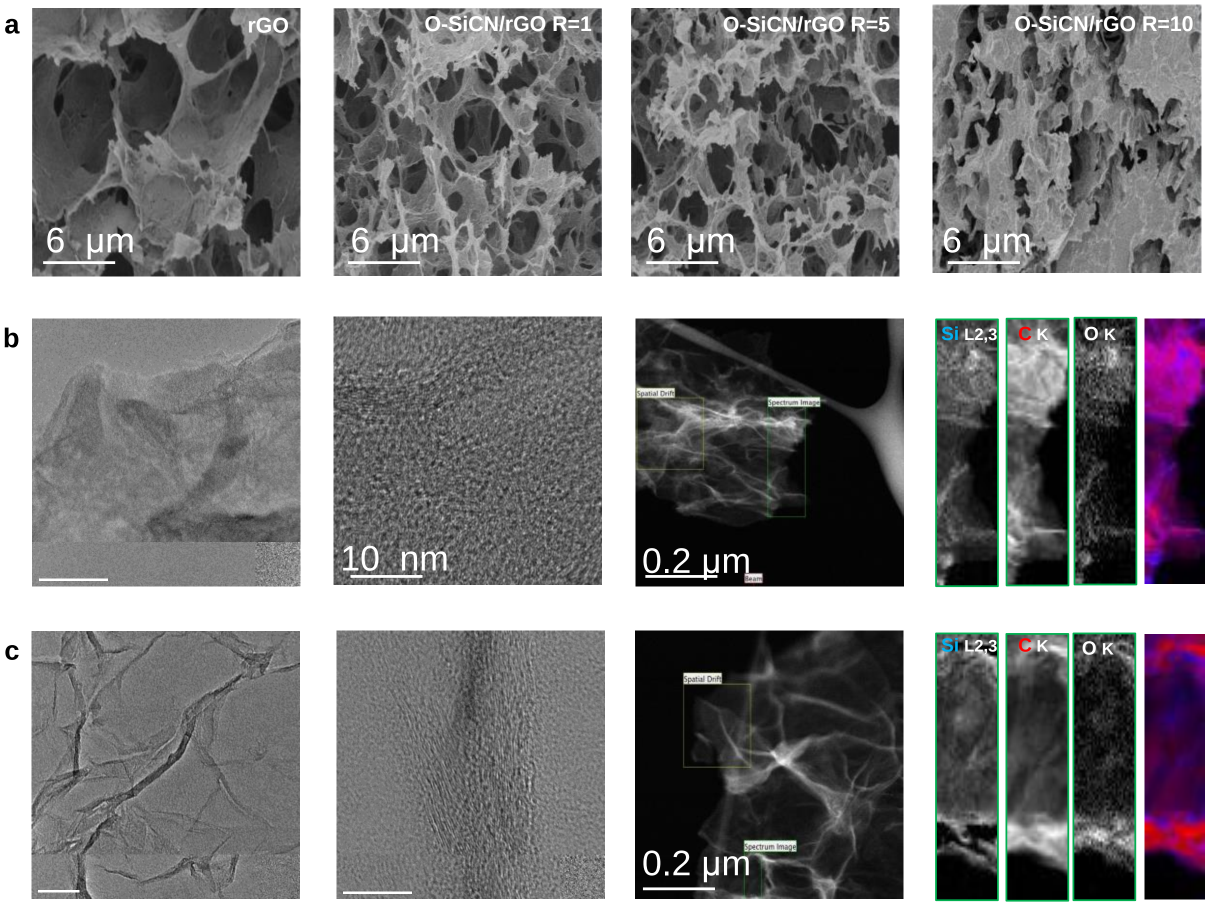

Figure S3 : (a) SEM images of rGO foam and O-SiCN/rGO composites at different ratios. (b,c) EELS with chemical maps for respectively O-SiCN/rGO and O-SiC/rGO composites with a 1:1 ratio.

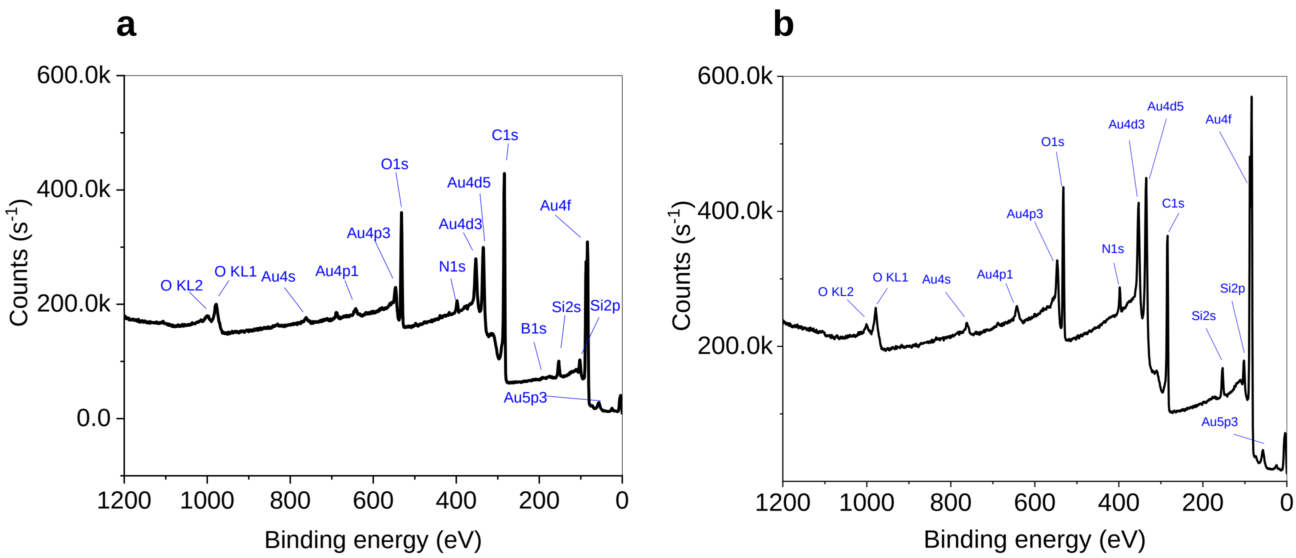

Figure S4: XPS survey spectra of (a) O-SiBCN/rGO and (b) O-SiCN/rGO composites. The spectra show that no metallic impurities are detected.

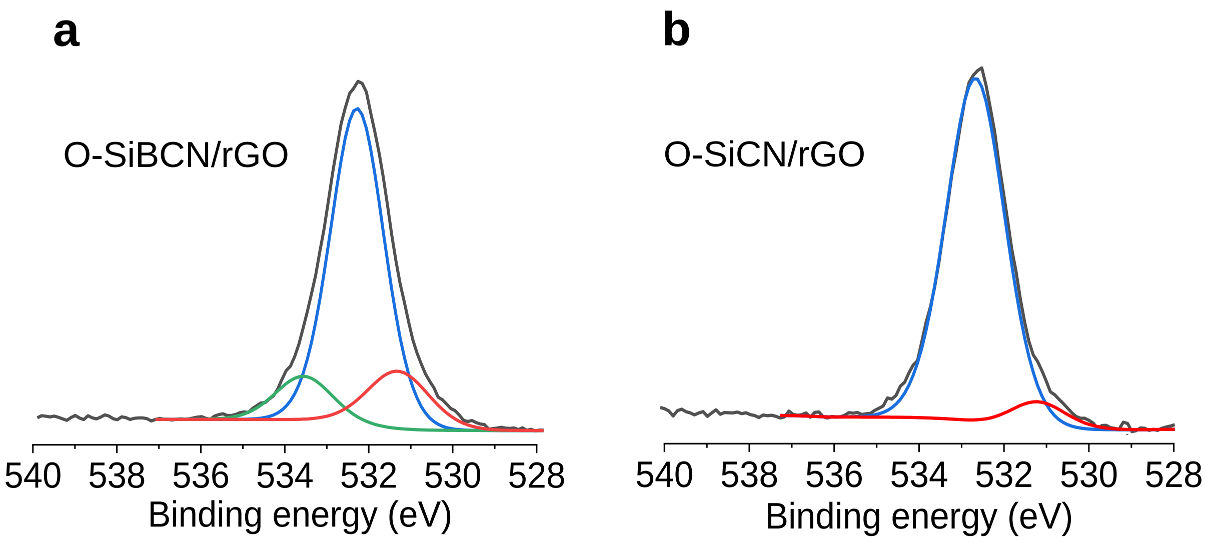

Figure S5: XPS spectra of O1s regions of (a) O-SiBCN/rGO and (b) O-SiCN/rGO composites.

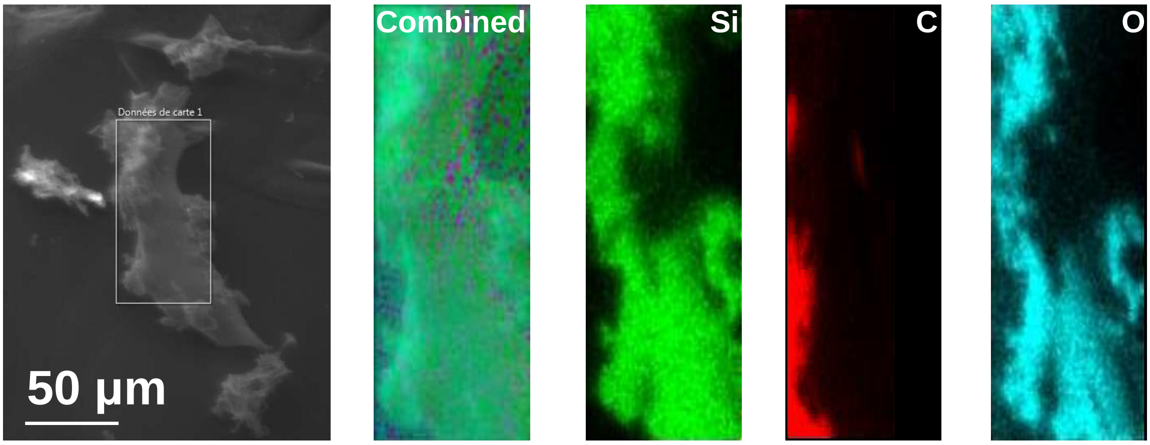

Figure S6 : SEM image and chemical maps on the TGA residue of O-SiBCN/rGO composite performed under air at 1000°C. The presence of Si and C elements demonstrates that the ceramic remained after the combustion of rGO.

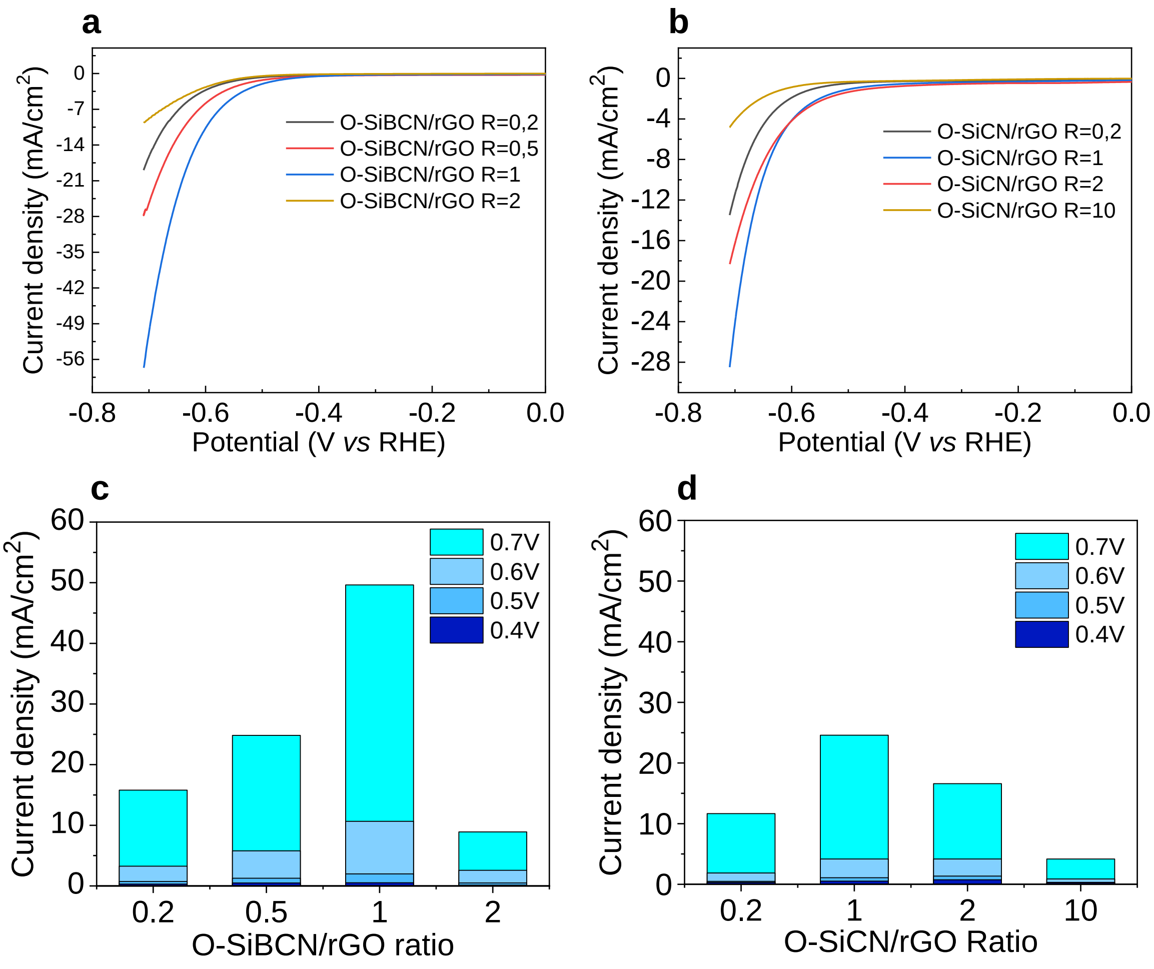

Figure S7: (a,b) Polarization curves of O-SiBCN/rGO and O-SiCN/rGO composites at different ratios, (c,d) Histograms representing the evolution of the current density at different potentials for the O-SiBCN/rGO and O-SiCN/rGO composites respectively. For both PDC/rGO systems we found an optimum of the catalytic activity for a PDCs/rGO 1:1 ratio.

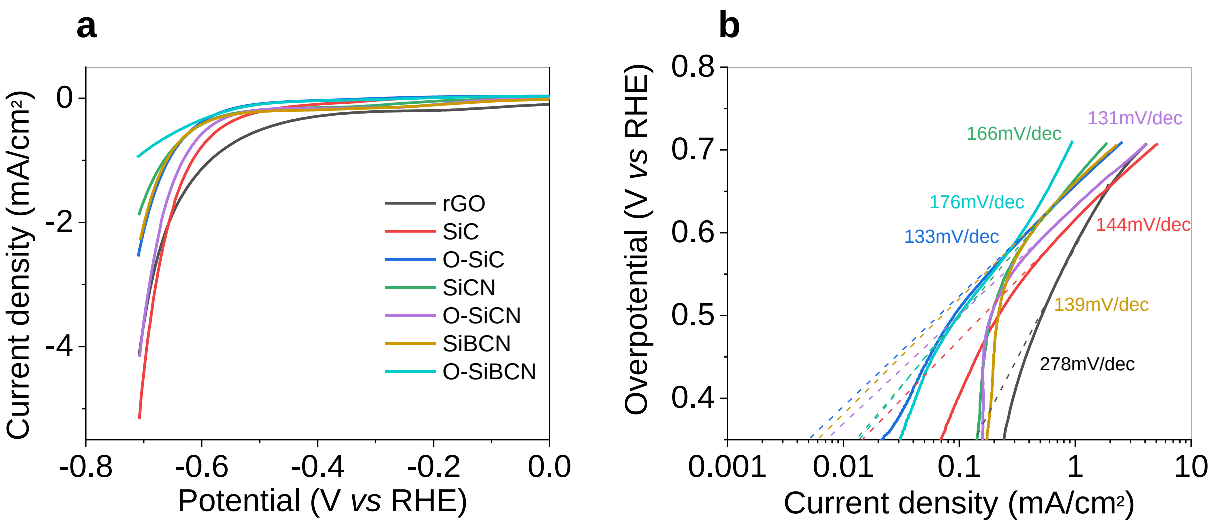

Figure S8 : (a) Polarization curves and (b) Tafel slopes of rGO, SiC, O-SiC, SiCN, O-SiCN, SiBCN, O-SiBCN catalysts.
